# New fluphenazine analogue with antimutagenic and anti-multidrug resistance activity—degradation profile and stability-indicating method

**DOI:** 10.1007/s00044-017-1944-4

**Published:** 2017-06-15

**Authors:** Agnieszka Sobczak, Artur Teżyk, Joanna Szyndlarewicz, Jan Ziarniak, Piotr Świątek, Wiesław Malinka

**Affiliations:** 1 Department of Pharmaceutical Chemistry, Poznan University of Medical Sciences, 6 Grunwaldzka Str., 60-780 Poznań, Poland; 2 Department of Forensic Medicine, Poznan University of Medical Sciences, 6 Święcickiego Str., 60-780 Poznań, Poland; 3 Department of Chemistry of Drugs, Wroclaw Medical University, 211 Borowska Str., 50-556 Wrocław, Poland

**Keywords:** Kinetics, Stability test, Degradation products, Phenothiazine analogue, HPLC, Mass spectrometry

## Abstract

Hydrochloride of 10-{2-hydroxy-3-[*N*,*N*-bis-(2-hydroxyethyl)amino]propyl}-2-trifluoromethylphenothiazine (Flu-A) is a analogue of neuroleptic fluphenazine. Flu-A exhibits anti-multidrug resistance, antimutagenic, proapoptopic, and cancer-chemopreventive activities in screening studies. To define identity, quality, and purity of new active substance it is necessary to develop a appropriate analytical method and to establish a degradation profile. Thus, a stability-indicating reversed-phase high-performance liquid chromatography method was developed and validated for quantitative determination of Flu-A in the presence of its degradation products generated under stress conditions. The compound was subjected to oxidation, photolysis, and degradation in aqueous solutions (neutral and acidic), and solid state according to the International Council for Harmonisation Guidelines. The method was also found to be suitable for intermediate and accelerated studies and for the evaluation of kinetic mechanism of Flu-A degradation in aqueous solutions (pH 5.1–7.5, 353 K). The structures of main potential degradation products were established using high-performance liquid chromatography-Electrospray Ionization-mass spectrometry method.

## Introduction

Cancer is very widespread in the modern world. According to World Health Organization in developed countries cancers are, after cardiovascular disease, the second leading cause of death. A major problem of modern oncology is the resistance of tumor cells to anticancer drugs (Klopman et al. 1997). The phenomenon of multidrug resistance (MDR) significantly impedes or even precludes effective, long-term chemotherapy in cancer. MDR frequently relates to simultaneous resistance of tumor cells to many medicinal substances, often of different chemical structure or different physicochemical properties (Klopman et al. 1997; Hendrich et al. 2003). Therefore, it seems appropriate to search inhibitors of the MDR process.

Analysis of clinical data showed that among patients with schizophrenia, treated with phenothiazines, the incidences of cancer are more seldom than in the rest of society (Fond et al. 2012). Phenothiazines, apart from their neuroleptic action, also exert different biological activities, which account for their cancer chemopreventive effect (Jaszczyszyn et al. 2012a). Unfortunately, limitations of phenothiazines use in the cancer treatment are caused by their serious side effects on the central nervous system (e.g., dyskinetic syndrome, parkinsonism, drowsiness, apathy, and depression). Hence, the structure of parent phenothiazines has been modified to obtain more hydrophilic analogues which exhibit similar antimutagenic/cancer-chemopreventive activity but fewer psychotropic effects. The modifications of neuroleptic phenothiazine structure consisted in introducing new substituents at the position 2 of the tricyclic phenothiazine ring system and changes in the length of the alkyl chain between the nitrogen atom at tricyclic skeleton and the terminal amine group in the side moiety (Gąsiorowski et al. 2003; Żyta et al. 2014).

**Fig. 1:**
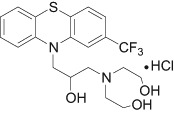
Structure of 10-{2-hydroxy-3-[*N*,*N*-bis-(2-hydroxylethyl)amino]propyl}-2-trifluoromethylphenothiazine hydrochloride (Flu-A)

Among the tested derivatives promising results were obtained for fluphenazine (Flu) analogue: 10-{2-hydroxy-3-[*N*,*N*-bis-(2-hydroxyethyl)amino]propyl}-2-trifluoromethylphenothiazine hydrochloride (Flu-A, Fig. 1) (Gąsiorowski et al. 2003; Żyta et al. 2014), which in screening studies exhibits antimutagenic, cancer-chemopreventive, and chemosensitizing activities. Flu-A inhibits P-glycoprotein-dependent MDR on the basis of various independent mechanisms, such as: an inhibition of protein kinase C activity, a fluidization of the structure of lipid membrane and a blocking of ligand binding sites within P-glycoprotein (Jaszczyszyn and Gąsiorowski 2006; Jaszczyszyn et al. 2012b). Moreover, Flu-A enhances apoptosis in genotoxically damaged lymphocytes 35% stronger than Flu (enhancement of apoptosis in tumor cells could be a desired mechanism of action of anticancer drugs in chemoprevention) (Gąsiorowski et al. 2003). The tested compound induces this process by stimulation of acidic sphingomyelinase and showed only a small effect on the activity of sphingomyelinase neutral (Jaszczyszyn et al. 2011). Analogue Flu-A also increased activity of caspase-3 in lympohocyte cultures genotoxically damaged in vitro with benzo[*a*]pyrene (Jaszczyszyn et al. 2009). The effect of Flu-A on the frequency of apoptosis was evaluated not only in cultures treated with benzo[*a*]pyrene, but also in undamaged cultures. However, this impact was not significant in undamaged lympohocyte what suggested that the enhancement of apoptosis could be specific, restricted to genotoxically damaged lympohocyte cultures. Simultaneously, fluphenazine derivative shows 60% lower cytotoxicity and 10% stronger antimutagenic effect than Flu (Gąsiorowski et al. 2003).

Before launching a new drug, it is necessary to define its physical and chemical properties in order to facilitate the understanding of mechanism of absorption, distribution, metabolism, excretion, and toxicity of the drug substance. Another important issue in the process of testing a chemical compound, as a potential candidate for a pharmaceutical formulation, is to evaluate its stability. Stability can be defined as the capacity of the active substance or drug product to sustain its identity, strength, quality, and purity throughout the retest or expiration period. Stability testing of drug substances or products includes changes in the quality of a drug product under the influence of environmental factors such as temperature, humidity, and light (Draft Guidance for Industry Stability Testing of Drug Substances and Drug Products 1998; ICH 2003). According to the International Council for Harmonisation (ICH) Guidelines Q1A (R2) in order to conduct stability tests should be develop and validate a stability-indicating analytical procedure (ICH 2003).

Based on the above mentioned the purpose of this studies was: (1) to develop and validate stability-indicating method for fluphenazine analogue (Flu-A); (2) to determine the effect of various environmental factors on its stability.

## Materials and methods

### Materials and reagents

The analogue of fluphenazine: 10-{2-hydroxy-3-[*N*,*N*-bis-(2-hydroxyethyl)amino]propyl}-2-trifluoromethylphenothiazine hydrochloride (Flu-A; C_20_H_24_ClF_3_N_2_O_3_S; Mol. mass 464.93g/mol) was synthesized at the Department of Chemistry of Drugs, Wroclaw Medical University, Poland (Żyta et al. 2014). The same batch was use in all studies. All other chemicals and solvents were of analytical or high-performance liquid chromatographic grade.

### Equipment and chromatographic conditions

The high-performance liquid chromatography (HPLC) method was developed and validated for forced degradation studies. The used HPLC system was composed of a Rheodyne Berkeley 7120 with a noose of 50 µL, an isocratic pump model liquid chromatography (LC)-61 Shimadzu and an SPD-6AV UV/VIS detector. The chromatographic conditions were as follows: an analytical column, Merck LiChrospher RP-18 (125 × 4 mm, 5 µm particle size); a mobile phase, the mixture of the solution containing 2.10 g/L of citric acid and 1.34 g/L of potassium chloride—acetonitrile (ACN) (70:30); flow rate, 1 mL/min; UV detection at 257 nm. The internal standard (I.S.) was propyl 4-hydroxybenzoate in a mixture of methanol and water (1:1) at a concentration of 0.16 mg/mL.

LC–mass spectrometry (MS) measurements were performed on a 1200 series liquid chromatograph coupled with a 6410B Triple Quad mass spectrometer (Agilent, USA). Separation was performed at 40 °C with a Poroshell 120 EC-18 column (3.0 × 75 mm, 2.7 µm, Agilent, USA). Mobile phases were: 0.1% formate buffer pH 3.2 [A] and 0.1% formic acid in acetonitrile [B]. The flow rate was 0.5 mL/min. The instrument was operated with electrospray ionization (ESI) source in the positive mode. MS conditions were: drying gas temperature (nitrogen), 300 °C; nebulizing gas flow, 8 L/min; nebulizing gas pressure, 40 psi; capillary voltage, 4 kV; fragmentor voltage, 50–250 V. The acquisition was carried out in the scan mode (*m*/*z* 50–500).

Photostability studies were carried out in a photostability chamber—the Atlas Suntest CPS + (Atlas, USA) which was equipped with temperature control system (25–100 °C), 1500 W air-cooled xenon lamps and with direct setting and control of irradiance in the wavelength range 300–800 nm (filter Solar ID65). The photostability tests were performed at 25 °C.

The pH of the tested solutions was measured with a potentiometric Hanna Instruments 110 pH-meter using an HI 1131 combination pH electrode at the respective experimental temperatures.

### Method validation

The HPLC method was validated according to the ICH Guidelines with regard to selectivity, linearity, precision, limit of detection (LOD), and limit of quantitation (LOQ).

### Selectivity

The selectivity of the HPLC method was evaluated by verifying the complete separation of Flu-A from its degradation products (DP) and the I.S. For this reason, the studied compound was subjected to thermal degradation in aqueous solutions at 363 K (24 h in 0.1 M; 36 h in 1 M and 72 h in 2 M HCl; 36 h and 14 days in water), oxidation (at 298 K, 6 h in 3%; then 3 h in 1.5% H_2_O_2_) and photodegradation.

### Linearity and LOD and LOQ

Linearity was established in the range from 0.02 to 0.32 mg/mL. Peak areas P_i_/P_I.S._ (P_i_ and P_I.S._—areas of Flu-A and I.S.) vs. Flu-A concentrations data were obtained by the least square linear regression analysis and used to calculate the calibration equations and correlation coefficients. The fourteen different concentrations were used for the linearity studies, and each sample was prepared in triplicate. The LOD and LOQ for the procedure were received directly from the calibration line using the formulas: LOD = 3.3*S*_*b*_/*a* and LOQ = 10*S*_*b*_/*a*, respectively, where *S*_*b*_ is the standard deviation of the intercept and *a* is the slope of the corresponding calibration curve.

### Precision

The measurement of precision was demonstrated by using the parameters of repeatability (intra-day) and intermediate precision (inter-day). In order to evaluate the repeatability of the method six replicate samples of Flu-A for three concentrations were analyzed. One of these concentrations was additionally determined (*n* = 6) on the second day in order to investigate the inter-day precision.

### Stability studies

Flu-A was exposed to different storage conditions: degradation in aqueous solutions (neutral and acidic), photodegradation, oxidation, dry and wet heat, as defined in the ICH Guideline Q1A (R2). Alkaline degradation was not performed because Flu-A, as a salt of an organic base, is insoluble in this environment. The I.S. was added to individual tested samples before the injection of the samples on HPLC column. All solutions for Flu-A degradation time *t* = 0 min, used in stress, intermediate and accelerated tests, were injected on HPLC at initial Flu-A concentrations of 0.20 mg/mL. All samples were protected from the light during the degradation stage, except for sample preparation time.

### Stress studies

Degradation in solutions was carried out at 363 K in water for 36 h and under acidic conditions (in 0.1 M HCl for 24 h, in 1 M HCl for 36 h and then in 2 M HCl for 72 h). After the degradation period, these solutions were neutralized (if necessary) and cooled in a mixture of ice and water.

Oxidative degradation was induced by storing the samples at room temperature (298 K) in 3 and 1.5% hydrogen peroxide for a period of 6 or 3 h, respectively.

Photostability of Flu-A compound in powder and in water solution was conducted in a quartz dish (in a layer less than 2 mm in thickness) and quartz cuvettes, respectively. All samples were inserted in a light cabinet (Atlas Suntest CSP+) and exposed to light for an overall illumination of 1.2 and then 6 million lux hours and an integrated near ultraviolet energy of 250 W/m^2^, at 25 °C. Control samples were protected from light with aluminum foils, placed in a light chamber and exposed concurrently.

### Intermediate and accelerated stability studies

Thermal stability and sensitivity to moisture of Flu-A in a solid state were investigated during its intermediate (relative air humidity ~ 65% ± 5%, 303 K, time: initial, 7 days, 1, 2, 3, 4, 6, 9, 12 months) and accelerated (~ 75% RH ± 5% RH, 313 K, time: initial, 7 days, 1, 2, 3, 4, 5, 6 months) studies. Degradation of Flu-A at 0% relative air humidity at 393 K was carried out for 12 months at different intervals, i.e. initial, 7 days, 1, 2, 3, 6 and 12 months.

In order to determine the stability of Flu-A in dry air, the vials containing the studied substance were immersed in sand baths, in heat chambers adjusted to 393 K.

The appropriate values of relative air humidity were obtained by using desiccators containing saturated solutions of suitable inorganic salts: sodium nitrite (~ 65% RH) or sodium chloride (~ 75% RH).

At individual time intervals, the vials with 2.5 mg of Flu-A were removed from the sand bath or from the desiccators placed in heat chambers, cooled to room temperature and their contents were dissolved in the mobile phase. The so-obtained solutions were quantitatively transferred into measuring flasks and diluted with a mobile phase to 10.0 mL.

### Kinetic procedures

The stability of Flu-A and its DP was investigated at 353 K in aqueous buffer solutions: acetate (pH = 5.1); phosphate (pH = 6.8); borate (pH = 7.5). The ionic strength, *µ* = 0.5 M, was adjusted for each solution by adding a calculated amount of sodium chloride (4 M). The pH value of the buffers was measured potentiometrically. The degradation was initiated by adding a dissolved sample of Flu-A to a buffer solution of specific pH equilibrated to the required temperature in a stoppered flask.

The starting Flu-A solutions (*t* = 0 min) were performed in the concentration of 0.5 mg/mL. At specified time intervals 0.5 mL of the above reaction solutions was collected and either neutralized if necessary (0.5 mL) or 0.5 mL of water was added. Then the samples were instantly cooled with a mixture of water and ice. 0.25 mL of I.S. solution was added to each sample and then they were analyzed.

### Identification of Flu-A products by HPLC-ESI-MS

The identification of the Flu-A degradation products was carried out by using the HPLC-ESI-MS method. The Flu-A samples were degraded under the following conditions:

- at 363 K in buffer solutions: acetate (pH = 5.1, 357 h), borate (pH = 7.5, 92 h),
- at 363 K in water (383 h),
- under oxidative agent (3% H_2_O_2_, 3 h, 298 K),
- under light (aqueous solution, dose 2700 kJ/m^2^, 3 h, 250 W/m^2^, 298 K),
- in a solid state (RH 0%, 6 months, 393 K; RH ~ 65%, 303 K, 9 months).

## Results and discussion

### Method development and optimization

The main objective of the chromatographic method was to separate the generated DP from the substrate and I.S. during stability studies. DP were co-eluted by using different aqueous components of mobile phases, such as dodecyl sodium, mixture of citric acid and potassium chloride or phosphate buffer, with various pH values (3.0–7.4), and organic modifiers, including ACN and methanol. Analysis of retention time, shape and intensity of peaks, resolution between analyte and its DP, ACN consumption while using different aqueous components of mobile phases indicated that the mixture of citric acid and potassium chloride was optimal. The use of methanol in a mobile phase caused poor and long elution of all compounds, thus acetonitrile was used in further studies. The change of ratio between the organic and water components, while maintaining a constant qualitative composition, resulted in a significant change in the retention times, the shape and intensity of the peaks. At higher acetonitrile content, the retention time of substrate (Flu-A) was shorter, and the peak was narrower, more symmetric and intensive. But on the other hand resolution between the DP peaks was the worst in these conditions. To improve the quality of chromatographic separation the influence of a mobile phase pH was investigated. It was observed that with the increase of pH the tailing of Flu-A peak was longer and also time of elution was longer, therefore pH equals to about 3 was chosen as optimal.

In conclusion, the best chromatographic separation was achieved on the column LiChrospher 100 RP-18 (125 mm × 4 mm, 5 μm) using a mobile phase (ACN—the mixture of 2.10 g/L of citric acid and 1.34 g/L potassium chloride) in proportions 30:70.

## Method validation

### Selectivity

**Fig. 2:**
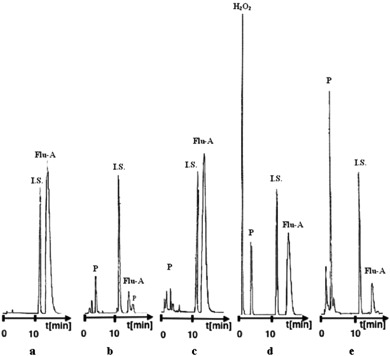
HPLC chromatograms of Flu-A degradation samples: **a** in water, 363 K, *t* = 0; **b** in water, 363 K, *t* = 14 days; **c** in HCl (2 M), 363 K, *t* = 72 h; **d** in H_2_O_2_ (1.5%), 298 K, *t* = 60 min.; **e** in solution, 298 K (55 046 lux h), *t* = 60 min.; where *I.S.*—internal standard, *P*—degradation products, *Flu-A*—10-{2-hydroxy-3-[*N*,*N*-bis-(2-hydroxylethyl)amino]propyl}-2-trifluoromethylphenothiazine hydrochloride

The results of stress tests indicated selectivity of the Flu-A quantitative method. In order to avoid the injection errors the I.S.—propyl 4-hydroxybenzoate was applied. The parent compound—Flu, was not used as a I.S. because it has a broad base as most of phenothiazines. The examples of typical chromatograms obtained for the pure Flu-A (in the presence of I.S.) and degraded samples are demonstrated in Fig. 2.

### Linearity and LOD and LOQ

The calibration curve for aqueous solutions of Flu-A was linear over the tested concentration range and the obtained correlation coefficient was 0.999 (*n* = 14) which shows an excellent correlation between the peak area and the analyte concentration. The equation of regression was as follows: *y* = (20.06 ± 0.48) · *x*. The value *b* *=* 0.091 calculated from *y* = *ax* + *b* was statistically insignificant, because the value *t*_*b*_ *=* *b*/*S*_*b*_ was lower than the critical one *t*_0.05_(12) (estimated for *f* = *n*−2 degrees of freedom (*n* = 14) at *p* = 0.05). The LOD and LOQ were 0.07 × 10^−1^ mg/mL and 0.02 mg/mL, respectively.

### Precision

The method had adequate repeatability because the relative standard deviation (RSD) for six repeated assays of samples was less than 2.0% for three different concentrations of Flu-A: 0.58% for *c* = 0.10 mg/mL, 0.85% for *c* = 0.20 mg/mL, 1.63% for *c* = 0.30 mg/mL. The RSD for inter-day precision was 1.10% for *c* = 0.20 mg/mL (*n* = 12).

### Stability studies

Stress tests of active substances or pharmaceutical products can help to identify the likely DP and to establish the decomposition pathways, which can be useful at the stage of development or modification of their chemical structure, manufacturing process, as well as their formulation or determining the storage conditions or validation of the stability-indicating analytical method. Moreover long-term, intermediate and accelerated stability studies allow evaluating the re-test period at quality monitoring or a shelf life of a drug product (Draft Guidance for Industry Stability Testing of Drug Substances and Drug Products 1998; ICH 2003).

### Stress, intermediate and accelerated stability studies

Flu-A was very stable in acidic solutions (HCl, 363 K) whereas in water degradation was observed (Table 1). In above cases color changes of tested solutions were observed (bright yellow or orange yellow color). The appearance of the color may indicate the presence of oxidation product—a radical cation. This is probable because in the presence of oxygen (from the air) in aqueous and buffered solutions of some phenothiazine derivatives, besides hydrolytic degradation the oxidation can occur (Pawełczyk et al. 1975; Underberg 1978). The thermal degradation of aqueous solutions of phenothiazines depends, first of all, on the kind of substituent at C2 and N10 positions (Pawełczyk et al. 1975).

**Table 1:** Results of stress, intermediate and accelerated degradation studies

Stress conditions	Period of study	Degree of degradation	ICH classification
Water, 363 K	36 h	sufficient degradation (37.9%)	unstable
Acidic solutions, 363 K			
0.1 M HCl	24 h	no degradation	very stable
1.0 M HCl	36 h	no degradation	
2.0 M HCl	72 h	9.3%	
Oxidation, 293 K			
3%	6 h	complete degradation	very unstable
1.5%	3 h	sufficient degradation (83.7%)	
Photodegradation (in aqueous solution)			
1.2 million lux hours, 250 W/m^2^, 298 K	21.8 h	complete degradation	photolabile
Photodegradation (solid state)			
1.2 million lux hours, 250 W/m^2^, 298 K	21.8 h	no degradation	photostable
Effect of temperature (solid state)			
393 K, 0% RH ± 5% RH	12 months	97.7%	–
Intermediate and accelerate studies			
303 K, 65% RH ± 5% RH	12 months	65.3%	–
313 K, 75% RH ± 5% RH	6 months	no degradation	–

Significant degradation of fluphenazine derivative was observed at 298 K under the oxidizing agents (6 h in 3% H_2_O_2_—total degradation; 3 h in 1.5% H_2_O_2_—sufficient decomposition) which classifies the compound as very unstable under an oxidative factor (Table 1).

Flu-A underwent photodegradation in aqueous solutions and was stable in the solid state on exposure to light (Table 1).

The significant degradation of Flu-A was observed at 0% relative air humidity at 393 K and ~ 65% RH at 303 K (intermediate studies). But during accelerated testing (~ 75% RH, at 313 K) the fluphenazine analogue was stable (Table 1). The phenomena of lower Flu-A stability under milder conditions of air humidity and temperature may be related to the oxidative properties of sodium nitrite, which was applied to obtain the suitable value of humidity. Thus, a different kind of salt should be used for stability studies at 65% RH.

### Kinetics studies

Based on the results of stress tests, the kinetic mechanism of Flu-A compound degradation was evaluated in a range of pH 5.1–7.5 at 353 K (at higher pH Flu-A compound underwent precipitation, and at lower pH its degradation time was significantly elongated).

**Fig. 3:**
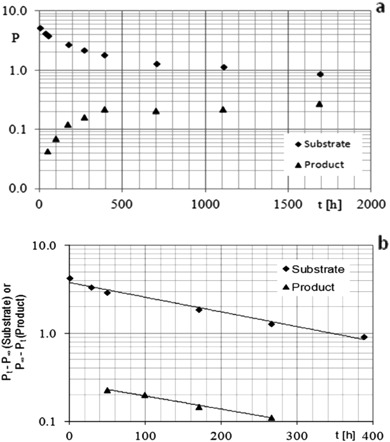
Plots of: **a** lnP = *f*(*t*) for reactions of Flu-A degradation and product A formation in acetate buffer, *c* = 0.10 M, (pH ~ 5.1, temp. 353 K); **b** ln(*P*_*t*_−*P*_∞_) = *f*(*t*) for Flu-A degradation reaction and ln(*P*_∞_−*P*_*t*_) = *f*(*t*) for product A formation in acetate buffer (*c* = 0.10 M, pH ~ 5.1, temp. 353 K)

The degradation of Flu-A in buffer solutions is a reversible first-order reaction relative to substrate concentration (Fig. 3).

During this study one main degradation product was analyzed. The concentration of product A in time interval from *t*_0_ to *t*_∞_ increased from 0 to *P*_∞_ (no further degradation of product A shows that the process is not a subsequent reaction) (Fig. 3a). Both processes: Flu-A degradation and product A formation can be described by the following equations:

$${\rm{ln}}\left( {{P_t} - {P_\infty }} \right) = {\rm{ln}}\left( {{P_0} - {P_\infty }} \right) - {k_{{\rm{obs}}}} \times t\quad \left( {{\rm{Flu}} \rm{-A}\ {\rm{degradation}}} \right)$$

$${\rm{ln}}\left( {{P_\infty } - {P_t}} \right) = {\rm{ln}}\left( {{P_\infty } - {P_0}} \right) - {k_{{\rm{obs}}}} \times t\quad \left( {{\rm{product}}\,{\rm{A}}\,{\rm{formation}}} \right)$$

where: *P*_0_, *P*_*t*_, *P*_∞_—the ratio of the peak area of Flu-A or product A to the peak area of I.S. at time zero, *t* and *t*_∞_, respectively; *k*_obs_—the observed first-order reaction rate constants; *t*—time (Fig. 3b). The values of reaction rate constants of Flu-A degradation and product A formation were compared using a parallelism test (Table 2). The results indicated that there are no statistically significant differences between them, which may suggest that product A is produced from Flu-A. But it does not exclude formation of product A by radicals. In this case rate constants of Flu-A and radicals degradation would be equal to rate constant of product A formation.

**Table 2:** Comparison of rate constants of Flu-A degradation and product A formation by using parallelism test

Slope of plots ln(*P*_*t*_−*P*_∞_) = *f*(*t*) of Flu-A degradation (*a* ± Δa)	Slope of plots ln(*P*_∞_−*P*_*t*_) = *f*(*t*) of product A formation (*a* ± Δa)	*t*_0_
Acetate buffer pH = 5.04, *c* = 0.10 M, 353 K		
−(3.85 ± 0.71) × 10^−3^ (h^−1^)	−(3.44 ± 0.99) × 10^−3^ (h^−1^)	*t*_*s*_ 2.447 > *t*_0_ 0.799
Phosphate buffer pH = 6.81, *c* = 0.10 M, 353 K		
−(2.54 ± 0.11) × 10^−3^ (h^−1^)	−(2.45 ± 0.47) × 10^−3^ (h^−1^)	*t*_*s*_ 2.262 > *t*_0_ 0.727
Borate buffer pH = 7.60, *c* = 0.14 M, 353 K		
−(1.29 ± 0.58) × 10^−2^ (h^−1^)	−(1.20 ± 0.21) × 10^−2^ (h^−1^)	*t*_*s*_ 2.306 > *t*_0_ 1.358

*t*_*s*_—value calculated of parallelism test

The catalytic effect was determined by measuring the rate of degradation of Flu-A at a constant pH (in all buffers), ionic strength (*μ* = 0.5 M) and temperature (353 K) (Table 3). Only the buffer concentration at a specific pH was different. The results obtained show (NaBO_2_, H_3_BO_3_) at concentrations applied demonstrate a catalytic effect. In acetate and phosphate buffers the rate constant (*k*_obs_) for the degradation of Flu-A is independent of their concentrations. It means that components like CH_3_COOH, CH_3_COONa, KH_2_PO_4_, Na_2_HPO_4_ do not catalyze the degradation reaction. This data can be useful at the selection of pharmaceutical excipients during formulation process.

**Table 3:** Observed rate constants *k* for the degradation of compound Flu-A in buffers at 353 K

Acetate buffer pH 5.1		Phosphate buffer pH 6.8		Borate buffer pH 7.5	
*c* (M)	(*k* ± Δ*k*) × 10^7^ (*s* ^−1^)	*c* (M)	(*k* ± Δ*k*) × 10^7^ (*s* ^−1^)	*c* (M)	(*k* ± Δ*k*) × 10^6^ (*s* ^−1^)
0.40	7.93 ± 1.03	0.20	9.61 ± 0.34	0.22	7.02 ± 1.04
0.30	9.98 ± 1.87	0.15	8.26 ± 0.66	0.19	6.30 ± 1.25
0.20	14.2 ± 3.56	0.10	7.08 ± 0.30	0.17	4.77 ± 0.56
0.10	10.7 ± 1.98	0.05	11.1 ± 1.48	0.14	3.60 ± 0.16
*t*_a_ = 2.929 < *t*_0.05=_4.403		*t*_a_ = 0.348 < *t*_0.05=_4.403		*t*_a_ = 7.229 > *t*_0.05=_4.403	

*t*_*a*_ < *t*_0.05_ buffer components do not have catalytic effect; *c*—buffer concentration

### MS analysis

**Fig. 4:**
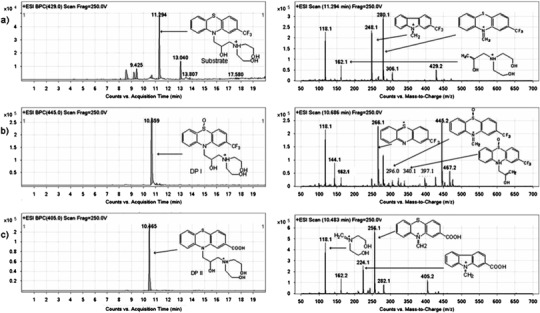
MS fragmentaion spectrum of substrate (Flu-A) **a** and its photodegradation products DP I **b** and DP II **c** (FV 250 V)

An LC-MS study was carried out to determine the molecular weight and structure of major Flu-A degradation products formed under oxidation, photodegradation and degradation both in solution (acidic and neutral medium) and a solid state. Flu-A degradation in all studied samples was incomplete, hence the peak at *t*_*R*_ = 11.3 min corresponding to Flu-A occurred at all obtained chromatograms. In the MS analysis at low fragmentor voltage (FV 50 V) of Flu-A a pseudo-molecular ion [M + H]^+^ at *m*/*z* 429 (Fig. 4a) and ammonium [M + NH_4_]^+^ and sodium [M + Na]^+^ adducts at *m*/*z* 445 and *m*/*z* 451, respectively were found. Moreover one or two major Flu-A degradation products were observed at chromatograms (DP I at 10.7 min; DP II at 10.5 min) (Fig. 4b,c). In all the above degradation conditions the most intensive product peak was observed at 10.7 min (DP I). Its mass spectrum exhibited a protonated molecular ion at *m*/*z* 445 (Fig. 4b) and ammonium (*m*/*z* 461) and sodium (*m*/*z* 467) adducts. Moreover, a pseudo-molecular ion [M + H]^+^ at *m*/*z* 405 (DP II) was found during photodegradation (Fig. 4c).

As it is known from literature that phenothiazine drugs undergo degradation under various factors (Nałęcz-Jawecki et al. 2008; Puzanowska-Tarasiewicz et al. 2009; Trautwein and Kümmerer 2012). They are easily oxidized by different chemical, photochemical, enzymatic (Puzanowska-Tarasiewicz et al. 2009), and electrochemical (Sobczak et al. 2015) agents often with a color change. The process of oxidation can proceed in several one-electron stages forming colored intermediate products—cationic radicals. Further oxidation leads to a generation of colorless sulphoxide. The first step of converting a substrate to radicals is relatively fast process opposite to the last stage of sulfoxide formation. Due to structure and lability of the radicals, they are not always recorded by every analytical methods. Stability of cationic radicals depends on a presence and a structure of substituents in positions 2 and 10, the nature of an oxidant applied, pH of solutions, the type and concentration of acid or buffer used, temperature and time of reaction (Borg and Cotziaz 1962; El-Gindy et al. 2002; Puzanowska-Tarasiewicz et al. 2005; Nałęcz-Jawecki et al. 2008; Puzanowska-Tarasiewicz et al. 2009). The hydrolysis of phenothiazines was also described in the literature (Pawełczyk and Marciniec 1974; Pawełczyk et al. 1975; Egorov 1998; Nałęcz-Jawecki et al. 2008).

The most frequently reported modifications of the molecule concerned S/N-oxidation, wherein N-oxides are formed mainly by oxidation of the nitrogen atoms present at the side chain at the 10-position. Oxidation at the nitrogen atom in phenothiazine ring is less likely to occur because substitution of an alkyl group at the 10-position causes oxidation at ring to be more difficult. Moreover transformation of the trifluoromethyl to a carboxylic group, elimination of amine side chains or dimerization can be also observed (Pawełczyk et al. 1975; Heyes 1982; Egorov 1998; Nałęcz-Jawecki et al. 2008; Trautwein and Kümmerer 2012).

Based on the above literature data connected with degradation of different phenothiazine derivatives, the molecular formula of DP I at *m*/*z* 445 could be defined as C_20_H_23_F_3_N_2_O_4_S and the corresponding structure could be attributed to any oxides of Flu-A: sulfoxides or N-oxides. For unambiguous identification of DP I higher FV (250 V) was used caused in-source fragmentation of compound. Fragmentation ions of the phenothiazine core combined with oxygen at *m*/*z* 296 and 340, which indicates oxidation at a sulfur atom in a phenothiazine ring, were observed. Furthermore, MS fragments at *m*/*z* 162 and 118 exclude oxidation at an aliphatic amine side chain. On this basis the product at *m/z* 445 could be attributed to a sulfoxide of Flu-A (DP I) (Fig. 4b).

Under light DP II formation were observed. MS fragmentation of DP II led to fragment ions *m*/*z* 224 and 256, which may indicate a transformation of the trifluoromethyl moiety to a carboxylic group (Fig. 4c).

DP I and II eluted at earlier retention time than their parent compound, which indicates their higher polarity. This observation may confirm the hypothesis that DP I (logP_calc._ = 1.90; the value calculated by ChemDraw program) is a sulfoxide of Flu-A (for Flu-A logP_calc._ = 2.93) and DP II is a carboxylic derivative of Flu-A (logP_calc._ = 1.57).

## **C**onclusions

In this paper stability of new fluphenazine analogue (Flu-A), which was designed to counteract the resistance of cancer cells to cytostatics, was investigated. Based on the results we obtained, it was demonstrates that Flu-A in solutions is sensitive to light and oxidative agents, so the substance should be protected before these factors in the future studies (preformulation process, testing on animal models etc.). Flu-A susceptibility to oxidation and photodegradation was confirmed by HPLC-ESI-MS and degradation profile obtained.

## Abbreviations

Flu: fluphenazine
Flu-A: analogue of fluphenazine
ICH: International Council for Harmonisation of Technical Requirements for Pharmaceuticals for Human Use
I.S.: internal standard
LOD: limit of detection
LOQ: limit of quantitation
MDR: multidrug resistance
RH: relative air humidity
RSD: relative standard deviation
